# Implementation of non-vitamin K antagonist oral anticoagulants in daily practice: the need for comprehensive education for professionals and patients

**DOI:** 10.1186/s12959-015-0046-0

**Published:** 2015-05-26

**Authors:** Hein Heidbuchel, Dana Berti, Manuel Campos, Lien Desteghe, Ana Parente Freixo, António Robalo Nunes, Vanessa Roldán, Vincenzo Toschi, Riitta Lassila

**Affiliations:** Hasselt University and Heart Center, Jessa Hospital, Stadsomvaart 11, Hasselt, 3500 Belgium; Department of Cardiovascular Medicine, University Hospital Gasthuisberg, University of Leuven, Leuven, Belgium; Department of Haematology, Thrombosis and Haemostasis, Unit- Centro Hospitalar do Porto, Porto, Portugal; Centre of Thrombosis and Haemostasis, Department of Transfusion Medicine, São João University Hospital, Porto, Portugal; Immunohaemotherapy Service, Centro Hospitalar Lisboa Norte and Hospital do SAMS-SBSI, Lisbon, Portugal; Haematology and Medical Oncology Unit, Hospital Universitario Morales Meseguer, University of Murcia, Murcia, Spain; Department of Haematology and Blood Transfusion, Thrombosis Centre, San Carlo Borromeo Hospital, Milan, Italy; Department of Haematology, Division of Coagulation Disorders, Cancer Centre, Helsinki University Central Hospital, Helsinki, Finland

**Keywords:** Anticoagulation clinic, Educational pathways, Non-vitamin K antagonist oral anticoagulant, Patient education

## Abstract

Non-vitamin K antagonist oral anticoagulants (NOACs) are increasingly used for the prevention and treatment of venous thromboembolism and for stroke prevention in patients with atrial fibrillation. NOACs do not require routine coagulation monitoring, creating a challenge to established systems for patient follow-up based on regular blood tests. Healthcare professionals (HCPs) are required to cope with a mixture of patients receiving either a vitamin K antagonist or a NOAC for the same indications, and both professionals and patients require education about the newer drugs. A European working group convened to consider the challenges facing HCPs and healthcare systems in different countries and the educational gaps that hinder optimal patient management. Group members emphasised the need for regular follow-up and noted national, regional and local variations in set-up and resources for follow-up. Practical incorporation of NOACs into healthcare systems must adapt to these differences, and practical follow-up that works in some systems may not be able to be implemented in others. The initial prescriber of a NOAC should preferably be a true anticoagulation specialist, who can provide initial patient education and coordinate the follow-up. The long-term follow-up care of patients can be managed through specialist coagulation nurses, in a dedicated anticoagulation clinic or by general practitioners trained in NOAC use. The initial prescriber should be involved in educating those who perform the follow-up. Specialist nurses require access to tools, potentially including specific software, to guide systematic patient assessment and workflow. Problem cases should be referred for specialist advice, whereas in cases for which minimal specialist attention is required, the general practitioner could take responsibility for patient follow-up. Hospital departments and anticoagulation clinics should proactively engage with all downstream HCPs (including pharmacists) to ensure their participation in patient management and reinforcement of patient education at every opportunity. Ideally, (transmural) protocols for emergency situations should be developed. Last but not least, patients should be well-informed about their condition, the treatment, possible risk scenarios, including the consequences of non-adherence to prescribed therapy, and the organisation of follow-up care.

## Background: non-vitamin K antagonist oral anticoagulants and the need for education

The well-documented limitations of vitamin K antagonists (VKAs) have led to the development of non-VKA oral anticoagulants (NOACs) that have more predictable pharmacological properties, do not require routine coagulation monitoring, depend less on patient and lifestyle factors, and have fewer restrictions in terms of diet and co-medications [1]. NOACs are changing clinical practice and are increasingly used for the prevention and treatment of venous thromboembolism (VTE) and stroke prevention in patients with non-valvular atrial fibrillation (AF).

Unlike VKAs, NOACs are not subject to regular dose adjustments [2]; however, their use requires changes to established systems for patient follow-up based on regular blood tests. The lack of a need for regular coagulation monitoring should not preclude regular contact with healthcare professionals (HCPs) to oversee the safe and effective management of NOAC therapy. Important checks include monitoring of renal and hepatic function, exclusion of thrombocytopenia or anaemia (especially in elderly patients), assessment of nuisance bleeding or other adverse events, confirmation of adherence to the prescribed medication and evaluation of other aspects of overall treatment and health, including optimal management of hypertension [2]. Because some patients are well managed on VKAs or have conditions for which NOACs are contraindicated (e.g. prosthetic heart valves), HCPs and healthcare systems are increasingly required to cope with patients receiving either a VKA or a NOAC for the same or different indications.

Anecdotal evidence suggests that many HCPs lack education on NOACs and are concerned about prescribing them. Additionally, specific management and follow-up protocols (e.g. for serious bleeding complications) are still in development. Although some patients receiving NOACs may not require frequent contact with an HCP, others, such as those with co-morbid conditions, renal impairment or a high risk of bleeding complications, should be seen more often. HCPs and patients must understand relevant aspects of the prescribed anticoagulant and the important differences between NOACs and VKAs. In many (but not all) countries, monitoring of patients receiving VKAs is usually performed at a dedicated outpatient clinic [3], and staff at these clinics serve well as focal disseminators of education on NOACs to HCPs and patients.

This document has been created to consider best practice for dissemination of education and the evolving role of the coagulation clinic in the NOAC era.

### Development of this document

A workshop was held in September 2013 involving European HCPs with expertise in anticoagulation from a range of specialties, including cardiologists, haematologists and specialist nurses from Belgium, Finland, Italy, Portugal, Slovenia, Spain and the United Kingdom. This working group was asked to consider the challenges facing HCPs and healthcare systems in their countries with respect to the introduction of NOACs, and the gaps in educational structures and settings that hinder or endanger the optimal management of patients receiving these drugs. The outputs of the discussions were taken forward to create this manuscript. Bayer HealthCare provided logistical support for the meeting.

### Current models of anticoagulant prescription and monitoring

To understand the flow of educational information within an anticoagulation management paradigm, it is first necessary to consider commonly used patient management models. These models are built primarily for patients receiving long-term VKA therapy and fall into two main categories: anticoagulation clinic coordination and coordination by general practitioners (GPs). In some systems, a hybrid model exists, in which unstable or complicated patients are seen in a hospital clinic and stable patients are managed by their GP. In all cases, the networking between these functions is critically important.

### Anticoagulation clinic model

Patients regularly attend a specialist outpatient clinic for international normalised ratio (INR) monitoring. Following prespecified algorithms, a nurse may take responsibility for INR interpretation and subsequent VKA dose adjustment, or may refer to the supervising consultant cardiologist or haematologist for atypical patients or scenarios. The nurse or consultant (or both) may take responsibility for educating the patient on their condition and treatment. Official anticoagulation clinics exist in several European countries, including Belgium, Italy, the Netherlands, Portugal, Spain, Slovenia, Sweden and the United Kingdom, but in many countries this service is decentralised.

### General practitioner model

In the absence of a dedicated outpatient clinic, responsibility for ongoing monitoring of patients receiving chronic anticoagulant therapy generally falls to GPs. GPs tend to be familiar with VKAs and have a detailed knowledge of their patients’ health and lifestyle. Practice nurses may take responsibility for routine INR monitoring in some cases. The GP model is used in Germany and to a variable extent in other countries. The risk of non-uniform management strategies and the lack of quality control may be a drawback of these services. A ptrecent study in the United States reported that the number of patients tested per GP practice was positively associated with INR time in therapeutic range (TTR) [4].

### Possible new models for long-term non-vitamin K antagonist oral anticoagulant care

Current systems face the need to integrate patients taking NOACs with those taking VKAs. Consultant cardiologists and haematologists have a strong knowledge base to support this integration but may not be at the front line of day-to-day patient interaction. Therefore, it is important that their knowledge is disseminated to nurses, GPs and other relevant HCPs. The models presented here draw on previous work by members of this consensus group [5]. Each model has advantages and drawbacks (Table 1) and would require adaptation to account for specific differences in country, regional and local healthcare systems

**Table 1:** **Advantages, disadvantages and requirements of proposed models for monitoring of patients taking non-vitamin K antagonist oral anticoagulants or vitamin K antagonists**

**Model**	**Initial prescriber**	**HCP responsible for routine follow-up**	**Advantages**	**Disadvantages**	**Requirements for NOAC integration**
Nurse-coordinated anticoagulation clinic	Hospital specialist	Nurse specialist	• Nurses well placed to coordinate contact with patients, the initial prescriber and other HCPs	• Requires well-educated expert nurses and resources for an anticoagulation clinic	• Determination of individual patient visit schedules
			• Nurses can take a holistic view (co-morbidities), make a full assessment and educate the patient	• Medico-legal liability issues	• Easy-to-manage patients could have primary contact through the GP; clinic could function as a coordinator and remote evaluator of care
			• Less intensive for the specialist, allowing them to focus fully on the treatment plan		• Non-medical as well as medical aspects and patient preference to be taken into account when considering whether to switch from VKA to NOAC
Nurse-assisted anticoagulation clinic	Hospital specialist	Cardiologist/haematologist, assisted by nurse	• Nurse does not require extensive anticoagulation expertise but can still organise patient visits and provide basic checks and education	• Resource- and time-heavy for specialist	• As above
GP coordinated, without anticoagulation clinic	Hospital specialist or specialist GP	GP	• Reduces pressure on hospital resources	• Increased pressure on GP resources	• GPs to maintain contact with patients at a frequency based on patient risks and preferences
			• GPs generally know their patients well	• GPs must be well trained in anticoagulation (NOACs as well as VKAs)	• Specialist department to be available to evaluate the patient at the GP’s request
			• Can perform home visits	• Good relationship/network needed between hospital departments and local community physicians	• GP may rely on the specialist for his/her own education – only well-educated GPs should be prescribers of NOACs

GP, general practitioner; HCP, healthcare professional; NOAC, non-vitamin K antagonist oral anticoagulant; VKA, vitamin K antagonist.

.

### Anticoagulation clinic models

#### Nurse coordinated

**Figure 1:**
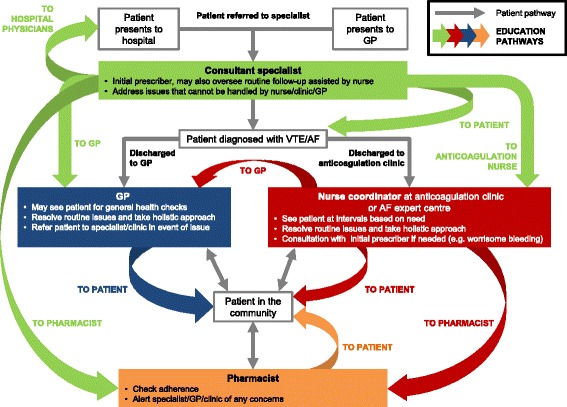
Patient flow and educational pathways in a model of long-term management of patients receiving NOACs. AF, atrial fibrillation; GP, general practitioner; HCP, healthcare professional; NOAC, non-vitamin K antagonist oral anticoagulant; VTE, venous thromboembolism.

Specialist anticoagulation nurses are well placed to drive the integration of NOACs into existing anticoagulation clinic structures [5]. In the nurse-coordinated model, a nurse coordinator, working according to standard operating procedures, takes responsibility for routine patient management in the anticoagulation clinic. The clinic is largely autonomous but remains under the supervision of consultant physicians, who see patients at predefined intervals and/or on referral from the nurse. The nurse keeps in contact with the patient, determines the frequency of clinic visits and may provide education to GPs, pharmacists and patients and their families about the medical condition and its treatment (Figure 1)

. Sufficient resources for training expert nurses and mechanisms for benchmarking and quality control are required. Pressure on resources could be eased by identifying easy-to-manage, low-risk patients receiving NOACs, with whom contact could be maintained primarily through the GP, with the nurse coordinator keeping in touch with the patient and controlling follow-up (e.g. by telephone with the patient or GP). In this case, the clinic functions as a structured coordinator of care, even when it is delivered by others.

#### Nurse assisted

The cardiologist/haematologist at the anticoagulation clinic may be the primary care coordinator [5] who manages the workflow and be the point of contact for the patient (Figure 1). This system requires a lower level of nurse expertise, although nurses can still assume much of the daily organisation and patient education. On the other hand, this model is time- and resource-heavy for the responsible physicians. Anticoagulation clinics in Italy, and some in Spain, use this system.

### General practitioner model

In systems lacking dedicated anticoagulation clinics, the GP is responsible for routine follow-up of patients taking NOACs (Figure 1). The GP can refer to the hospital consultant in the event of problems that require specialist intervention, such as a thromboembolic event or major bleeding episode. GP-based follow-up can ease the demands on hospital resources, and the GP may even perform home visits for elderly patients or others for whom office visits are difficult. Many GPs are currently undereducated about NOAC therapy and may consider that the lack of routine coagulation monitoring means that no follow-up is needed, or may be unwilling to allocate time and resources to educate and evaluate patients receiving NOACs. A good relationship and networking opportunities between cardiology/haematology departments and GPs is, therefore, vital.

GPs should maintain contact with their patients at a frequency advised by the initial prescriber based on the patient’s risk profile and preferences. The GP may rely on the hospital cardiology/haematology department for their own education on NOAC therapy. In some countries (such as Belgium, Finland, Germany, Portugal and Spain), GPs are allowed to initiate anticoagulant treatment. This may not be ideal unless they have specialist knowledge of NOACs, but even so, the mechanisms for quality control remain uncertain.

### Important considerations for incorporation of non-vitamin K antagonist oral anticoagulants into all models

**Figure 2:**
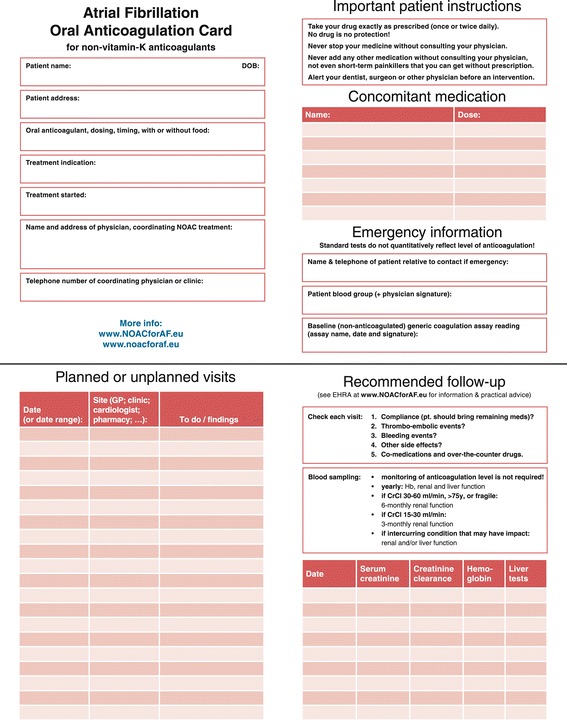
Adapted European Heart Rhythm Association (EHRA) proposal for a universal anticoagulation card for patients receiving non-vitamin K antagonist oral anticoagulants. Pharmacy has been included in the list of visit sites (page 2), and the results of baseline (prior to initiating anticoagulation) generic coagulation assays and the date of the test have been added to the emergency information (page 4), as indicated. A printable version of this card is available as a supplementary file. Adapted from Heidbuchel *et al. Europace* 2013;15:625–51 [2]. GP, general practitioner; CrCl, creatinine clearance; NOAC, non-vitamin K antagonist oral anticoagulant.

**Figure 3:**
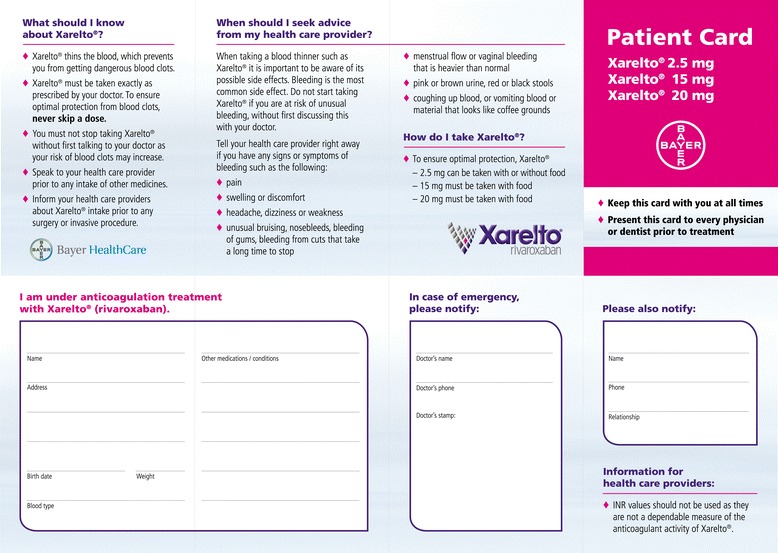
Example of a drug-specific patient anticoagulation card for rivaroxaban. Copyright Bayer HealthCare, reproduced with permission.

Patients receiving NOACs may not need to interact with an HCP as often as those taking VKAs, but nevertheless, contact frequency should be based on individual medical needs. Extensive contact may not be required for the majority of patients who are in generally good health, have a limited potential for relevant drug interactions and have a low or moderate risk of bleeding, provided that they receive appropriate education and are expected or known to have good treatment adherence. In patients with a high risk of bleeding, anaemia, thrombocytopenia or declining renal or hepatic function, or likely or suspected suboptimal adherence, the responsible HCP should see the patient directly on a regular basis [6]. To keep track of relevant information and remind patients of important aspects of their therapy, the European Heart Rhythm Association (EHRA) has proposed a universal NOAC patient anticoagulation card (Figure 2; printable version in supplementary material) [2], and companies have produced drug-specific versions (example in Figure 3)

. Information on the patient’s baseline (non-anticoagulated) readings for relevant generic or specific coagulation assays (e.g. activated partial thromboplastin time or dilute thrombin time [Hemoclot®] for dabigatran, and prothrombin time or anti-Factor Xa assays for direct Factor Xa inhibitors), and the date on which these tests were performed, could also be added to the card or the electronic laboratory file of the patient. This is important if these tests are used to check for the presence or absence of a NOAC effect in an emergency.

For stable patients with AF receiving VKAs, the European Society of Cardiology (ESC) considers a TTR of 70% to constitute good management [7]. However, in real life, an INR range of 2.0–3.0 is often not attained [8,9], and in some randomised clinical studies in which the INR was monitored rigorously, the mean TTR was only 55–65% [10-13]. HCPs should consider whether their patient really is well managed on a VKA; considering the broad indications for NOAC therapy, it is likely that most patients are candidates for switching to NOACs, provided they do not have contraindications. For a patient with poor adherence, the HCP responsible may feel more comfortable with regular monitoring, but constant dose adjustments due to irregular INRs may actually contribute to the problem, and switching to a fixed-dose NOAC may simplify treatment and improve adherence. The possible psychological impact on patients caused by the loss of monitoring visits (e.g. social contact for elderly patients and reassurance that their medication is working) may be overcome by regular visits for other necessary tests or an overall health check. However, the decision to switch or not should be taken by the HCP and the patient in tandem, based on both being informed about the treatment choices available.

To harmonise procedures, a centralised database of all NOAC- and VKA-anticoagulated patients should be created and made available to all relevant HCPs within the local system. This database should provide information on diagnosis, drug and dose regimen and anticipated duration of treatment, alongside agreed standard management protocols. Data on INR measurements and TTR would also be useful to inform decisions about switching. The information within such a system could be controlled by the relevant overseeing specialist department.

#### Opinion statements

- Where the healthcare structure allows, long-term management of patients receiving anticoagulation can be efficiently handled by a centralised (anticoagulation) clinic. As an alternative, GPs or specialist nurses with appropriate training and experience can take responsibility, with specialist support and supervision
- Initiation of NOAC treatment should be restricted to GPs who receive specialist training and should otherwise be avoided
- Practical incorporation of NOACs into healthcare systems must take into account national, regional and local variations in practice as well as available resources; anticoagulation clinics may be best suited to handle routine medical and educational tasks and networking between specialties
- Problem cases and scenarios need to be referred to an expert facility run by coagulation specialists

### Flow of education to support non-vitamin K antagonist oral anticoagulant implementation: needs, responsibilities and support materials

Patients receiving anticoagulants have contact with different HCPs (including, but not limited to, those specified in this document) at different time points during their treatment. Each of these HCPs has a responsibility to educate and/or reinforce patient education. HCPs must have the appropriate level of knowledge and be able to communicate it effectively in terms that the patient can understand. This requires that information migrates from clinical consultants (usually the initial prescribers) through the layers of the healthcare network to reach the patient. This can occur through regular HCP education meetings in the anticoagulation clinic, adoption of national guidelines, support from patient anticoagulation associations and provision of public information.

### The initial prescriber

The clinical diagnosis of a thromboembolic condition and the initial decision to prescribe anticoagulant treatment is best done by a specialist (e.g. a cardiologist, neurologist, geriatrician or haematologist) rather than by a GP or other physician without training or experience of anticoagulant treatment. Firstly, specialists have access to the latest clinical data and insights and, therefore, have the expertise to make the initial prescribing decision (and plan the length of treatment); secondly, they can provide patient education and disseminate relevant information to other HCPs involved in downstream care (Table 2, Figure 1)

**Table 2:** **Educational needs and responsibilities for healthcare professionals responsible for patients receiving non-vitamin K antagonist oral anticoagulant therapy**

**The initial prescriber**	**The anticoagulation clinic nurse**	**The general practitioner**	**The pharmacist**	**The patient**
*Needs*	*Needs*	*Educational needs*	*Educational needs*	*Educational needs*
• Access to the latest data and expert opinion via colleagues, academic literature, and internal and external meetings and congresses	• To receive regular updates on current best practice from the cardiology/haematology department (as detailed above); nurse coordinators also need access to academic literature and the chance to attend national coagulation nursing meetings	• Ongoing updates (6-monthly in the first 1.5 years and annual thereafter) from specialists on evolving treatment options for VTE/AF; these could be provided online or by email, or via an in-hospital training day, and tied to professional development	• Ongoing (annual or ad hoc) updates from anticoagulation clinic nurses and/or GPs regarding the loco-regional organisation of structured anticoagulant care (who is responsible for what, which lines of communication are available, etc.) and questions to ask the patient when filling a prescription (adherence – check blister pack)	• Basic knowledge of their condition, treatment and the importance of adherence
• Simple flowcharts outlining recommended indications, dose adjustment and follow-up	• Clear and concise guides on how to perform follow-up actions (SOPs), including clear instructions on how and when to contact their supervising physician, GPs, other HCPs and patients themselves throughout follow-up	– GPs are likely to be familiar with VKAs but less so with NOACs, and they will need to receive education on the differences between NOACs and other agents, and important aspects of ongoing care of patients receiving NOACs, including patient education	*Educational responsibilities*	• Awareness of what to do if an adverse event occurs, and ability to differentiate between minor and major events
*Responsibilities*	• Software to assist in these tasks	– What to do in different scenarios; what constitutes an emergency/which issues should be referred via a routine appointment to the coagulation clinic/specialist department	• To patients:	• Regular contact with an HCP responsible for follow-up at a frequency dependent on individual risk assessment
• Provide education to other hospital departments and community GPs on treatment options for VTE/AF (regular updates)	• To the patient (at each visit)	– Support with posters/checklists/desk note reminder cards, references to online sources of information	– Ensure that appropriate questions are asked of the patient when they collect their medication, based on the indication for use and the type of patient (elderly, with renal impairment, etc.) and medication (NOAC, VKA, etc.)	• Materials and tools to assist with ongoing education and therapeutic adherence
– Hospital meetings, including specific training days for GPs if practicable (could link to professional development)	*Responsibilities*	– Provide patient leaflets and checklists to GP offices	– Ensure that patients are not prescribed contraindicated co-medications	*Educational responsibilities*
– Support with posters, checklists, desk note reminder cards that HCPs can take away and use; provide links to online sources of information	– Continual reinforcement of key educational messages about the anticoagulant they are taking (NOAC or VKA) at each visit (frequency of visits different for each patient based on health and risk factors for thrombosis/bleeding as discussed above)	*Educational responsibilities*	– Add follow-up information to the patient’s NOAC card	• To take a proactive role in their own treatment
• Provide education and training to anticoagulation clinic nurses (at least 6-monthly updates)	– Informal assessment of patient understanding (e.g. can they name their condition and explain why they have to take an anticoagulant, what the dose is, what signs of bleeding they should be looking out for, etc.?)	• To the patient	• Notify the coagulation clinic and/or GP of any concerns about the patient	
– This could take the format of a session co-chaired by a senior nurse from the clinic or a GP experienced in NOAC use plus the specialist to give two different perspectives (practical and scientific)	– Support with printed leaflets and checklists, educational posters in the office, etc.	– In the absence of an anticoagulation clinic: follow-up with patients receiving anticoagulants (NOACs or other) at regular intervals (frequency dependent on risk) to remind them of the important aspects of their medication and what to do if they are concerned about an aspect of their health relating to their treatment		
– Topics	• To GPs	– As an adjunct to an anticoagulation clinic: take the opportunity at any patient visit for any reason other than anticoagulation to remind them of the important points regarding their anticoagulant treatment (adherence – check blister pack to make sure all doses taken)		
• Which patients are suitable for NOACs (e.g. stable condition, good renal function, knowledgeable about treatment, having a carer in charge of medication) vs which are not (or less) suitable (e.g. confused, elderly, likely to be non-compliant, with contraindications) – support with case studies	– Periodical (3- to 6-monthly?) contact to check on individual patients	• To pharmacists (ad hoc communication):		
• Fundamental aspects of each NOAC vs heparin/VKAs (e.g. predictable pharmacology, drug interactions, etc.)	• Those requiring only infrequent visits to the anticoagulation clinic	– Pass on educational information on questions to ask the patient before issuing a repeat prescription (this interaction could be mediated by the patient NOAC card)		
• Practical guides (e.g. based on EHRA advice) on topics such as taking a full bleeding history, calculating risk score (CHA_2_DS_2_-VASc, HAS-BLED, etc.), switching, appropriate laboratory tests and how to interpret them	• Follow-up on patients who have missed scheduled clinic visits	– Remind about important aspects of pharmacology, drug interactions, etc.		
• ‘What to do if…’ guidance, including identification of potential emergency scenarios (serious bleeding event, e.g. head injury, major surgery required) vs minor problems (e.g. nose bleed, minor surgical procedure) and whom to contact in each case	• Receive information on changes of patient status that may lead to an intensification of follow-up (e.g. anaemia, thrombocytopenia, new concomitant medication that may increase the risk of bleeding, exclusion of hypertension)	– Send posters/checklists; encourage pharmacist to get in contact if they have concerns		
• Latest information on patient adherence and how it can be improved; which practical tools they can use and offer to patients	– Reminders about key aspects of anticoagulants (NOAC and VKA), e.g. pharmacology, drug interactions, etc., and warning signs to look out for in patients receiving them (e.g. bleeding, vomiting, bruising)			
– Smartphone/tablet apps and other online tools could be used in case physical meetings cannot be held	– Support with posters/checklists/desk note reminder cards, references to online sources of information			
• Education of the patient about their condition and how it will be managed	• To the pharmacist			
– First discussion at point of initial prescription	– Periodical (annual?) contact to remind pharmacists about questions they should ask the patient when filling a repeat prescription for any anticoagulant (e.g. ask the patient to present his/her NOAC card; the pharmacist could also complete a line on the follow-up page of the NOAC card, indicating how many drug doses have been delivered or any other important information), and, specifically for NOAC vs LMWH/VKA, whom to contact in case of concerns			
• Emphasise patient understanding of their condition and the drug they are taking (NOAC or VKA), and the importance of correct adherence to the prescribed regimen – use language that the patient can understand	– Key information on pharmacology and drug interactions			
• Support with printed leaflets and checklists that the patient can take home – these can include QR codes/online links, but should be physical copies so that patients can keep them in a convenient place and have constant access to them	– Support with posters/checklists/desk note reminder cards, references to online sources of information			
– Second discussion to reinforce messages before discharge				
• Warning signs to look out for (e.g. anaemia, thrombocytopenia, hypertension, bleeding, vomiting, bruising)				
• Importance of keeping follow-up appointments with coagulation clinic/GP				
• Why adherence is important				
– Support with printed leaflets and checklists as above				
– Provision of NOAC card to the patient				

AF, atrial fibrillation; EHRA, European Heart Rhythm Association; GP, general practitioner; LMWH, low molecular weight heparin; NOAC, non-vitamin K antagonist oral anticoagulant; QR, quick response; SOP, standard operating procedure; VKA, vitamin K antagonist; VTE, venous thromboembolism.

; and thirdly, they also gather centralised data on complications and their management, which are useful for the wider healthcare community. Ideally, education should be based on national guidelines written by representatives of those disciplines that are most likely to encounter emergency situations involving NOACs.

After discharge, the initial prescriber often ceases to have primary responsibility for patient follow-up but should remain available to consult in the case of difficulties. The responsible HCP should be aware of the scenarios, such as trauma and acute inflammatory and gastrointestinal diseases (poor absorption or vomiting), that should trigger a specialist consultation. In countries such as Italy, the initial prescriber, in most cases the haematologist working in the anticoagulation clinic, is also responsible for follow-up of patients receiving NOACs.

Many cases of VTE or AF are diagnosed in the emergency room. Patients entering the system via this route may never actually be admitted to an inpatient department, but instead may be discharged with a prescription for an oral anticoagulant. It would be preferable for these patients to transition first via an anticoagulation clinic.

#### Opinion statements

- The initial prescriber should preferably be an experienced specialist
- The initial prescriber is also responsible for initial patient education and for educating other HCPs about thrombotic conditions, anticoagulant treatment and duration, and risk scenarios
- The initial prescriber may or may not have primary responsibility for further follow-up, but should be available for consultation and to gather information on complications

### Anticoagulation clinic nurse

It is the opinion of this writing group that the follow-up and continuing education of all patients receiving long-term NOAC or VKA anticoagulation in the community is best performed by a specialist anticoagulation clinic, headed either by a specialist nurse(s) (nurse coordinated) or by a cardiologist/haematologist supported by nurses (nurse assisted). In the NOAC era, in patients treated for VTE, an ideal time for a first follow-up visit may be at the point that patients transition from parenteral to dabigatran therapy or from the more intensive doses of apixaban (after 7 days of treatment) or rivaroxaban (after 21 days). During follow-up, the role of the anticoagulation nurse becomes wider than basic INR monitoring and includes other tests, such as calculating creatinine clearance, taking a holistic view of patient care and providing education to patients and other HCPs (Table 2, Figure 1). This education includes knowledge about unstable situations, such as acute illness, starting or stopping co-medications, trauma and management of major and even minor (e.g. tooth extraction) surgery. A single clinic is likely to be responsible for patients receiving NOACs and those receiving VKAs and must address the different requirements of all patients.

**Figure 4:**
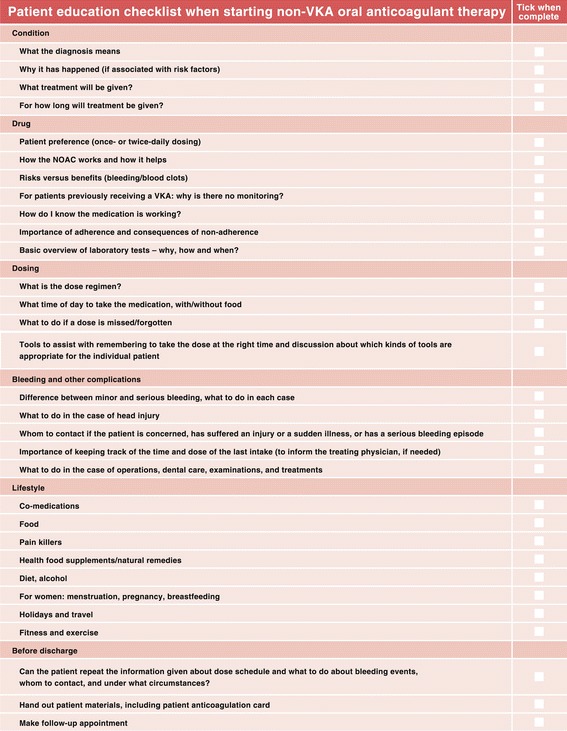
Basic educational checklist for healthcare professionals when managing patients starting non-vitamin K antagonist oral anticoagulant therapy. NOAC, non-vitamin K antagonist oral anticoagulant; VKA, vitamin K antagonist.

Evidence suggests that patients with AF have a poor level of education about their disease, its severity and its treatment [14], and that improving their knowledge is crucial to the success of anticoagulant therapy [15,16]. The University of Maastricht in the Netherlands has pioneered a software-based approach to nurse-led coordination of care. Patient data are entered into the software program to generate a risk score and an advised course of therapy, based on guidelines. The nurse-led approach, overseen by a supervising cardiologist, was shown to reduce cardiovascular hospitalisations and deaths [17], and to correlate with an improvement in patients’ AF-related knowledge over time [18], compared with standard cardiologist-led care. Centres could consider developing their own software or adapting existing programs to fit their protocols. A sample checklist for patient education is shown in Figure 4

. Materials such as booklets, validated websites, smartphone apps and short videos that provide important information to patients in an accessible manner are extremely useful.

#### Opinion statements

- A specialist coagulation nurse is well placed to take overall responsibility for patient education and to coordinate follow-up with other HCPs
- Coagulation nurses require specialist education, examples of best practice, and access to simple and effective tools to assist with patient education
- Specific software tools that help the nurse with systematic assessment of the patient and workflow guidance should be developed and shared

### The general practitioner

A GP is often involved in the initial diagnosis of a thromboembolic disorder but should be encouraged to swiftly refer all suspected cases to the specialist hospital department. This is especially important in patients with suspected pulmonary embolism and those with renal or hepatic impairment. It is possible that GPs consider they have a responsibility to prescribe anticoagulation therapy. However, if an established care network with rapid back-up from a specialist centre is available, they may be more willing to refer the prescribing decision to a specialist, which will improve overall patient care.

The GP may be the most frequent point of contact for a patient receiving a NOAC after discharge and can take responsibility for follow-up of patients deemed at low risk. In the absence of an anticoagulation clinic, the role of GPs in patient education assumes greater importance and they will have the main responsibility for long-term patient follow-up (Table 2, Figure 1). Hospital departments and anticoagulation clinics should proactively engage with GPs and provide them with education and training on the management of patients taking NOACs.

A GP education programme for NOACs should have a practical focus and include obligatory training sessions with real-life problems/clinical cases that the GPs can submit for discussion, as well as simple guidance and algorithms. GPs should know whom to contact in the specialist centre in case of questions or concerns about specific patients.

#### Opinion statements

- In the absence of an anticoagulation clinic, GPs are likely to take responsibility for the management and ongoing education of patients receiving NOACs
- Even when a clinic exists, patients considered at low risk of bleeding may not be required to attend regularly; in this case, the GP may take responsibility for follow-up
- GPs should reinforce educational messages regarding anticoagulation with patients at every opportunity, even if the patient is visiting for another reason
- Hospital departments or anticoagulation clinics should proactively engage with GPs and provide them with education on NOACs

### The pharmacist

Pharmacists have an important (and so far underestimated and underused) role in the monitoring of patient adherence to NOAC treatment. In particular, they can check that patients understand the dose and regimen, as well as reinforcing general educational messages (Table 2, Figure 1). Hospital departments or anticoagulation clinics should proactively engage with pharmacists to ensure they can effectively participate in the management of patients receiving anticoagulants. Some countries have national databases that support the pharmacist in evaluating patient adherence. Further research into the development of such systems is warranted.

#### Opinion statements

- Pharmacists are important in checking patient adherence
- Hospital departments or anticoagulation clinics should proactively engage with pharmacists and provide them with education on NOACs, including drug interactions

### The patient

In the NOAC era, with less regimented HCP contact, patients must increasingly take responsibility for their own understanding of their condition and treatment. Primarily, they need to have a basic knowledge of why they are being treated, how their treatment works, the importance of adherence and what to do if an adverse event occurs. Differentiation between a minor event that can be self-managed (e.g. a nose bleed) and one that should prompt them to contact an HCP or go to the emergency department is required. Even if a patient is not regularly attending a GP or anticoagulation clinic, the responsible HCP should keep in regular contact, e.g. with a brief telephone call. Patients should be able to ask questions, and family members should be included if appropriate, such as in the case of patients with diminished self-responsibility. Materials can be provided to remind patients of important information, and these should be tailored to the individual patient. For example, a calendar can help to remind patients to take their treatment, but some patients may find a pill box with days of the week, or a reminder on their mobile telephone, to be more effective. Several organisations also offer online patient support websites, including the EHRA (http://www.afibmatters.org/), the AF Association in the UK (http://www.atrialfibrillation.org.uk/) and Anticoagulation Europe (http://www.anticoagulationeurope.org/). Ideally, some of this content could be translated into local languages. Patients must be aware of the importance of their patient card (Figure 2).

#### Opinion statements

- Therapy is likely to be enhanced by proactively involving the patient in the management of their condition; this requires that the appropriate level of information is given using appropriate language, and confirmation that the provided information has been understood by the patient
- Educational messages should be reinforced with all patients by all HCPs at every opportunity

## Conclusions

Incorporation of NOACs into healthcare systems must take account of national, regional and local variations, and it is recognised that some systems may not be able to incorporate all the suggestions in this document. The initial prescriber of a NOAC should preferably be a specialist, who may handle problem and emergency cases as well as proactively engage with all downstream HCPs to ensure that they participate effectively in patient management and reinforce patient education. The initial prescriber has to consider involvement in the education of other HCPs and coordination of patient follow-up. The long-term care of patients may be managed through an anticoagulation clinic and/or specialist nurses, under guidance and supervision. Coordination by GPs who are trained in NOAC use is an alternative. GPs and pharmacists should be involved in the follow-up pathways that are coordinated by anticoagulation clinics or specialists. Last but not least, the patient should be well informed about their condition, treatment, possible risk situations and the organisation of follow-up care. Only a patient with insight into their condition will be fully adherent to therapy and follow-up and have a successful outcome.
